# Supplementary material for: Influence of maternal use of tenofovir disoproxil fumarate or zidovudine in Vietnamese pregnant women with HIV on infant growth, renal function, and bone health
Source: PLoS One. 2021 Apr 29;16(4):e0250828. doi: 10.1371/journal.pone.0250828 (PMC8084453; doi:10.1371/journal.pone.0250828)
Supplement: Attachment — Submitted filename: PONE-D-21-05310_reviewer (1).pdf [file pone.0250828.s002.pdf]

PLOS ONE

Influence of maternal use of tenofovir disoproxil fumarate or zidovudine in Vietnamese pregnant women with HIV on infant growth, renal function, and bone health

--Manuscript Draft--

**Manuscript Number:**	PONE-D-21-05310
**Article Type:**	Research Article
**Full Title:**	Influence of maternal use of tenofovir disoproxil fumarate or zidovudine in Vietnamese pregnant women with HIV on infant growth, renal function, and bone health
**Short Title:**	Maternal use of tenofovir vs. zidovudine and infant transient rickets in Asian population
**Corresponding Author:**	Ei Kinai<br>Tokyo Medical University: Tokyo Ika Daigaku<br>Tokyo, Tokyo JAPAN
**Keywords:**	Tenofovir disoproxil fumarate, pregnancy, infant, transient rickets, renal tubular dysfunction, bone health
**Abstract:**	Tenofovir disoproxil fumarate (TDF) is still widely prescribed for HIV-1 pregnant women, despite its renal and bone toxicity. Although TDF-exposed infants often show transient growth impairment, it is not clear whether maternal TDF cause transient infantile rickets via maternal/fetal renal dysfunction especially in Asian populations. This prospective observational study was conducted in Vietnam and involved pregnant HIV-infected women treated with TDF-based regimen (TDF group) or zidovudine-based regimen (AZT-group). At birth, 3, 12, and 18 months of age, we measured body length, weight, head circumference, serum alkaline phosphatase (ALP), creatinine, calcium, phosphorus, urine-β2-microglobulin (U-BMG), percentage of tubular reabsorption of phosphate (%TRP), and radiographic wrist score for rickets. Age-adjusted multivariate linear regression models were fit to evaluate the association of TDF/AZT use during pregnancy with fetal renal function and bone health. The study included 63 mother-infant pairs (TDF group=53, AZT group=10). In the mothers, detectable U-BMG (>252 μg/L) was observed more frequently in the TDF- than AZT group (89 vs 50%, p<0.001), but other renal/bone parameters were similar. In infants, maternal TDF use was not associated with growth impairment, renal dysfunction, or abnormal bone findings, but with a slightly higher ALP levels (p=0.019). However, shorter length was associated with maternal AZT (p=0.021), and worse radiographic scores were associated with LPV/r (p=0.024). In Vietnamese population, TDF use during pregnancy was not associated with infant transient rickets, growth impairment, or renal function, though it was associated with mild maternal tubular impairment. Maternal AZT and LPV/r influenced infant growth and bone health, though further studies are needed to confirm this finding.
**Order of Authors:**	Ei Kinai
	Hoai Dung Thi Nguyen
	Ha Quan Do
	Shoko Matsumoto
	Moeko Nagai
	Junko Tanuma
	Kinh Van Nguyen
	Thach Ngoc Pham
	Shinichi Oka
**Additional Information:**	
**Question**	**Response**
**Financial Disclosure**	This research was supported by the Japan Initiative for Global Research Network on Infectious Diseases from the Japan Agency for Medical Research and development,

Powered by Editorial Manager® and ProduXion Manager® from Aries Systems Corporation

Enter a financial disclosure statement that describes the sources of funding for the work included in this submission. Review the submission guidelines for detailed requirements. View published research articles from *PLOS ONE* for specific examples.<br><br>This statement is required for submission and **will appear in the published article** if the submission is accepted. Please make sure it is accurate.<br><br>**Unfunded studies**<br>Enter: *The author(s) received no specific funding for this work.*<br><br>**Funded studies**<br>Enter a statement with the following details:<br>• Initials of the authors who received each award<br>• Grant numbers awarded to each author<br>• The full name of each funder<br>• URL of each funder website<br>• Did the sponsors or funders play any role in the study design, data collection and analysis, decision to publish, or preparation of the manuscript?<br>• **NO** - Include this sentence at the end of your statement: *The funders had no role in study design, data collection and analysis, decision to publish, or preparation of the manuscript.*<br>• **YES** - Specify the role(s) played.<br><br>* typeset	AMED. During the study period, antiretrovirals for Vietnamese patients were provided under the financial support of the U.S. president's emergency plan for AIDS relief (PEPFAR).<br>The funders had no role in study design, data collection and analysis, decision to publish, or preparation of the manuscript.
**Competing Interests**<br><br>Use the instructions below to enter a competing interest statement for this submission. On behalf of all authors, disclose any competing interests that could be perceived to bias this work—acknowledging all financial support and any other relevant financial or non-financial competing interests.<br><br>This statement is required for submission and **will appear in the published article** if the submission is accepted. Please make	E.K. has received honoraria from Gilead Sciences, ViiV Healthcare, and MSD. S.O. received honoraria from MSD, Janssen Pharmaceutical and Gilead Sciences, and research grants from MSD, ViiV Healthcare, Gilead Sciences. The remaining authors declare no conflict of interest.<br>This research was supported by the Japan Initiative for Global Research Network on Infectious Diseases from the Japan Agency for Medical Research and development, AMED. During the study period, antiretrovirals for Vietnamese patients were provided under the financial support of the U.S. president's emergency plan for AIDS relief (PEPFAR).

Powered by Editorial Manager® and ProduXion Manager® from Aries Systems Corporation

sure it is accurate and that any funding sources listed in your Funding Information later in the submission form are also declared in your Financial Disclosure statement.<br><br>View published research articles from *PLOS ONE* for specific examples.<br><br>**NO authors have competing interests**<br><br>Enter: *The authors have declared that no competing interests exist.*<br><br>**Authors with competing interests**<br><br>Enter competing interest details beginning with this statement:<br><br>*I have read the journal's policy and the authors of this manuscript have the following competing interests: [insert competing interests here]*<br><br>* typeset	
**Ethics Statement**<br><br>Enter an ethics statement for this submission. This statement is required if the study involved:<br><br>• Human participants<br>• Human specimens or tissue<br>• Vertebrate animals or cephalopods<br>• Vertebrate embryos or tissues<br>• Field research<br><br>Write "N/A" if the submission does not require an ethics statement.<br><br>General guidance is provided below. Consult the submission guidelines for detailed instructions. **Make sure that all information entered here is included in the Methods section of the manuscript.**	The study involved human participants. The study was conducted in accordance with the ethical guidelines for medical and health research involving human subjects (revised Feb.28, 2017), the Ministry of Health, Labor and Welfare, Japan. The study was approved by the Institutional Review Board of National Center for Global Health and Medicine (NCGM), Tokyo, Japan (NCGM-G-002030-00), National Hospital of Tropical Diseases (NHTD), Hanoi, Vietnam (18/HDDD-NDTU), and National Hospital of Obstetrics and Gynecology (NHOG), Hanoi, Vietnam (936/CN-PSTW). Each participant provided a written informed consent after a thorough explanation of the purpose of the study and potential outcome.

Powered by Editorial Manager® and ProduXion Manager® from Aries Systems Corporation

**Format for specific study types**<br><br>**Human Subject Research (involving human participants and/or tissue)**<br>• Give the name of the institutional review board or ethics committee that approved the study<br>• Include the approval number and/or a statement indicating approval of this research<br>• Indicate the form of consent obtained (written/oral) or the reason that consent was not obtained (e.g. the data were analyzed anonymously)<br><br>**Animal Research (involving vertebrate animals, embryos or tissues)**<br>• Provide the name of the Institutional Animal Care and Use Committee (IACUC) or other relevant ethics board that reviewed the study protocol, and indicate whether they approved this research or granted a formal waiver of ethical approval<br>• Include an approval number if one was obtained<br>• If the study involved *non-human primates*, add *additional details* about animal welfare and steps taken to ameliorate suffering<br>• If anesthesia, euthanasia, or any kind of animal sacrifice is part of the study, include briefly which substances and/or methods were applied<br><br>**Field Research**<br><br>Include the following details if this study involves the collection of plant, animal, or other materials from a natural setting:<br>• Field permit number<br>• Name of the institution or relevant body that granted permission	
**Data Availability**<br><br>Authors are required to make all data underlying the findings described fully available, without restriction, and from the time of publication. PLOS allows rare exceptions to address legal and ethical concerns. See the PLOS Data Policy and FAQ for detailed information.	Yes - all data are fully available without restriction

A Data Availability Statement describing where the data can be found is required at submission. Your answers to this question constitute the Data Availability Statement and **will be published in the article**, if accepted.<br><br>**Important:** Stating 'data available on request from the author' is not sufficient. If your data are only available upon request, select 'No' for the first question and explain your exceptional situation in the text box.<br><br>Do the authors confirm that all data underlying the findings described in their manuscript are fully available without restriction?	
**Describe where the data may be found in full sentences. If you are copying our sample text, replace any instances of XXX with the appropriate details.**<br><br>• If the data are **held or will be held in a public repository**, include URLs, accession numbers or DOIs. If this information will only be available after acceptance, indicate this by ticking the box below. For example: *All XXX files are available from the XXX database (accession number(s) XXX, XXX.).*<br>• If the data are all contained **within the manuscript and/or Supporting Information files**, enter the following: *All relevant data are within the manuscript and its Supporting Information files.*<br>• If neither of these applies but you are able to provide **details of access elsewhere**, with or without limitations, please do so. For example:<br><br>*Data cannot be shared publicly because of [XXX]. Data are available from the XXX Institutional Data Access / Ethics Committee (contact via XXX) for researchers who meet the criteria for access to confidential data.*<br><br>*The data underlying the results presented in the study are available from (include the name of the third party*	Data are available from the Ethics Committee of the National Center for Global Health and Medicine, Tokyo, Japan. for researchers who meet the criteria for access to confidential data.

and contact information or URL).<br>• This text is appropriate if the data are owned by a third party and authors do not have permission to share the data.<br><br>* typeset	
Additional data availability information:	

Powered by Editorial Manager® and ProduXion Manager® from Aries Systems Corporation

Manuscript
Click here to access/download;Manuscript;TDF and rickets-Manuscript (plain text) 210217-2.docx

Influence of maternal use of tenofovir disoproxil fumarate or zidovudine in Vietnamese pregnant women with HIV on infant growth, renal function, and bone health

Ei Kinai, MD, PhD[1,2], Hoai Dung Thi Nguyen, MD[3], Ha Quan Do, PhD[4], Shoko Matsumoto, PhD[2], Moeko Nagai, MSc[2], Junko Tanuma, MD, PhD[2], Kinh Van Nguyen, MD, PhD[3] , Thach Ngoc Pham, MD, PhD[3], and Shinichi Oka, MD, PhD[2]

1 Department of Laboratory Medicine, Tokyo Medical University, Tokyo, Japan

2 AIDS Clinical Center, National Center for Global Health and Medicine, Tokyo, Japan

3 National Hospital for Tropical Diseases, Hanoi, Vietnam

4 National Hospital of Obstetrics and Gynecology, Hanoi, Vietnam

Corresponding author:

Prof. Ei Kinai, MD, PhD

Department of Laboratory Medicine, Tokyo Medical University

6-7-1, Nishishinjuku, Shinjuku-ku, Tokyo 160-0023, Japan. Phone: +81 (3) 3342-6111. Fax: +81 (3) 3340-5448. Email: ekinai@tokyo-med.ac.jp

Short title: Maternal use of tenofovir vs. zidovudine and infant transient rickets in Asian population

Key words: Tenofovir disoproxil fumarate, pregnancy, infant, transient rickets, renal tubular dysfunction, bone health

Abstract

write it out

Tenofovir disoproxil fumarate (TDF) is still widely prescribed for HIV-1 pregnant women, despite its renal and bone toxicity. Although TDF-exposed infants often show transient growth impairment, it is not clear whether maternal TDF cause transient infantile rickets via maternal/fetal renal dysfunction especially in Asian populations. This prospective observational study was conducted in Vietnam and involved pregnant HIV-infected women treated with TDF-based regimen (TDF group) or zidovudine-based regimen (AZT-group). At birth, 3, 12, and 18 months of age, we measured body length, weight, head circumference, serum alkaline phosphatase (ALP), creatinine, calcium, phosphorus, urine-β2-microglobulin (U-BMG), percentage of tubular reabsorption of phosphate (%TRP), and radiographic wrist score for rickets. Age-adjusted multivariate linear regression models were fit to evaluate the association of TDF/AZT use during pregnancy with fetal renal function and bone health. The study included 63 mother-infant pairs (TDF group=53, AZT group=10). In the mothers, detectable U-BMG (>252 μg/L) was observed more frequently in the TDF- than AZT group (89 vs 50%, p<0.001), but other renal/bone parameters were similar. In infants, maternal TDF use was not associated with growth impairment, renal dysfunction, or abnormal bone findings, but with a slightly higher ALP levels (p=0.019). However, shorter length was associated with maternal AZT (p=0.021), and worse radiographic scores were associated with LPV/r (p=0.024). In Vietnamese population, TDF use during pregnancy was not associated with infant transient rickets, growth impairment, or renal function, though it was associated with mild maternal tubular impairment. Maternal AZT and LPV/r influenced infant growth and bone health, though further studies are needed to confirm this finding.

I wouldn't use first person

lower?

usage?

INTRODUCTION

Tenofovir disoproxil fumarate (TDF) is still widely used for treatment of HIV infection. For pregnant women, TDF is the only available option of two nucleoside reverse transcriptase inhibitors, since the recommendation by the World Health Organization (WHO) in 2013 [1]. However, TDF is known to be nephrotoxic [2] mainly causing proximal tubular dysfunction[3,4], which results in renal loss of phosphorus with a subsequent subsequent decrease in bone mineral density [5,6]. This mechanism was confirmed in a study that showed tenofovir alafenamide (TAF), a novel prodrug, reduced TDF-related nephrotoxicity, resulting in significant improvement in not only renal tubular function but also in bone mineral density [3].

Based on the severe toxicity of TDF on renal function and bone health, the safety of TDF in pregnant woman is a matter of concern. Especially, given TDF during pregnancy can theoretically cause nutritional rickets during early neonatal life due to maternal renal loss of phosphate in late pregnancy [7]. Previous studies in rhesus macaques showed that TDF administration in neonates and infants resulted in severe rickets [8]. In human, most of the large studies suggested that maternal TDF was not associated with infant growth impairment worldwide [9-11]. However, some longitudinal cohort studies interestingly showed that TDF-exposed infants presented with

This is a long sentence. I would recommend shortening and rewording

transient shorter length within a few months after birth than non TDF-exposed infant, which was overcome by 1-2 year of age [9,12]. In addition, while bone mineral

be specific. What site.

density (BMD) was lower in TDF-exposed infants at the first 4 weeks after birth [13], it became comparable level at 12 months after birth [14]. These findings suggested that maternal TDF can cause transient nutritional rickets due to maternal renal loss of

phosphate~~s~~, which can be overcome by nutritional replacement after birth. Furthermore, there is limited information on the influence of maternal TDF on infant growth in Asian population [14, 15], ~~although they are~~ despite being considered ~~to be~~ vulnerable to TDF-associated nephrotoxicity due to the small body size [16]. The present study was designed to evaluate whether maternal TDF can cause transient nutritional rickets ~~tentatively~~ by impairing infant growth, renal function, or bone health in Vietnamese pregnant women with HIV.

MATERIALS AND METHODS

Sample population

This prospective observational study started in December 2016 and finalized December 2019. Vietnamese HIV-infected pregnant women who were already on anti-retroviral therapy (ART) were recruited at the National Hospital of Obstetrics and Gynecology (NHOG) before the third trimester. Each participant provided a written informed consent after a thorough explanation of the purpose of the study and potential outcome. ~~We applied the following~~ included the following inclusion criteria; 1) HIV-infected pregnant women, 2) free of acute AIDS-defining diseases, and 3) on treatment at the time of study entry with either TDF- or zidovudine (AZT)-based regimen. The inclusion criteria of each treatment groups ~~are~~ were the use of TDF- or AZT- based regimen; 1) later than 26 gestational weeks and 2) for more than 8 weeks. We also applied the following exclusion criteria; 1) HIV-infected infants (confirmed by HIV antibody test at 18 month of age), 2) preterm infants (<35 gestational weeks or <2000 g of birth weight), 3) multiple pregnancy (twins, triplets, or more), 4) congenital disorders (e.g.,

failure to thrive, rickets or renal impairment). Since sex-specific standard normal values for height, weight, head circumference and laboratory values are not available for Vietnamese newborn/infants, we also recruited HIV-non-infected mothers and their infants in this study (not shown in Fig. 1).

The study was approved by the Institutional Review Board of National Center for Global Health and Medicine (NCGM), Tokyo, Japan (NCGM-G-002030-00), National Hospital of Tropical Diseases (NHTD), Hanoi, Vietnam (18/HDDD-NDTU), and National Hospital of Obstetrics and Gynecology (NHOG), Hanoi, Vietnam (936/CN-PSTW).

Data collection

The enrolled pregnant women were asked to visit the NHTD at 32-36 gestational week for blood and urine tests on the viral load, CD4 cell count, and renal function (Fig. 1). The NHOG provided delivery care support, checked whether newborns met inclusion criteria, measured the body length, weight, and head circumference at birth,

What is this? First time seeing it.

and recorded baseline clinical information, such as APGAR score. After the delivery, the mother-infant pairs, who conformed the study inclusion and exclusion criteria, visited the NHTD at 3, 12, and 18 months after delivery for maternal and infantile measurements of various physical parameters and laboratory tests. Specifically, the infant length, weight and head circumference, and serum alkaline phosphatase (ALP), creatinine, calcium, phosphorus, urine-β2-microglobulin (U-BMG), percentage of tubular reabsorption of phosphate (%TRP), and wrist radiographic score for rickets were obtained at these three visits. Serum 25 (OH) Vitamin D level was determined at

3 months of age.

Radiographic scoring for nutritional rickets

Infant wrist radiographic score for nutritional rickets was determined at 3, 12, and 18
what do you mean, wrist part?
months of age, using the wrist part of a widely adapted 10-point scoring system[17].
a of a a a
Briefly, grade 0 represents normal wrist, grade 1 represents widened growth plate and
a
irregularity of metaphyseal margin without concave cupping, and grade 2 represents
metaphyseal concavity with fraying of margins. The maximum score is 4, with 2 for
both the radius and ulna
~~each of the two wrist bones (radius and ulna~~). Each radiograph was scored
independently by a trained physician at the National Center for Global Health and
Medicine, who was blinded to clinical information.

Statistical analysis

The clinical characteristics of the mothers and the infant baseline data were compared between the TDF and AZT groups using the chi-square test for nominal data and by
outcomes
the Mann-Whitney test for continuous parameters. The infant growth ~~parameter~~s
(length, weight, and head circumference) of the TDF and AZT groups were compared by two-way ANOVA at different ages. The prevalence of detectable U-BMG (>252 μg/L) and abnormal radiographic score for rickets in the two treatment groups were compared by the chi-square test. We also used linear multiple regression analyses to determine the relationship between growth parameters and the laboratory test values, and confounding factors other than maternal use of TDF and infant age, where maternal use of LPV/r, maternal age, CD4, and viral load, and gestational age, and

infant age were set as the independent variables, and the estimates, 95% confidence interval (95% CI) were calculated. For U-BMG, multivariate logistic analysis was conducted where the presence of detectable level of U-BMG was set as the dependent variable with the same independent variables as in multiple regression analyses. All statistical analyses were performed using The Statistical Package for Social Sciences ver. 23.0 (SPSS, Chicago, IL).

RESULTS

Sample population

A total of 67 HIV-infected pregnant women (57 TDF-treated women and 10 AZT-treated women) were enrolled in this study before the third trimester. Of those, 63 mother-infant pairs (TDF group, n=53, AZT group, n=10) who delivered at the NHOG met the study criteria (Fig. 1). ~~With regard to the non-HIV infected mother-infant pairs, although more than 30 mothers were recruited, only 2 mother-infant pairs were enrolled in the present study (Table 1).~~ Both of them provided maternal demographic and laboratory data and infant physical data at birth, however, none visited for further infant follow-up (not shown in Fig. 1). The median maternal age was 32 years [interquartile range (IQR) 8.0], and median body weight during 32-36 gestational
median
weeks were 57.5 kg (IQR 11.0), suggesting a non-pregnant body weight of less than
which is
50 kg, similar to the majority of Vietnamese women (Table 1). There were no
do not abbreviate the first time you present this
significant differences in age, body weight, VL and CD4 cell count between the TDF
recommends
and AZT groups. Based on the 2013 WHO guidelines, which ~~recommended~~ the combination of TDF+3TC+EFV for all HIV-infected patients, including pregnant

women, 53 of the 63 (84%) pregnant women received TDF-based regimen, while 49 of the 63 (78%) received TDF+3TC+EFV. Furthermore, LPV/r was used by 5 of the 10 (50%) mothers of the AZT group, but only by 7 of the 53 (11.1%) of the TDF group.

Effects of TDF on maternal laboratory tests

Laboratory tests showed a tendency for higher levels of serum creatinine (0.59 vs. 0.53 mg/dL, p=0.276), lower concentrations of serum phosphorus (3.1 vs. 3.6 mg/dL, p=0.358), and higher percentage of mothers with low %TRP (38.7% vs. 20.0%, p=0.309) in the TDF group, compared with the AZT group, respectively. Furthermore, a significantly larger proportion of mothers of the TDF group had high U-BMG compared with the AZT group (89% vs. 50%, p<0.001) (Table 1).

Effects of TDF and AZT on newborn physical characteristics

The majority of infants were born at 39 weeks of gestation and none showed clinical b~~B~~ody length and head circumference at birth were smaller in the AZT group compared with the TDF group [49.2 vs. 46.4 cm, p=0.061 (body length), and 34.0 vs. 32.5 cm, p<0.001 (head circumference) in TDF and AZT group, respectively]. In Vietnam, all infants of HIV-infected mothers are treated with nevirapine, but not AZT, during the first 6 weeks of life. The 25 (OH) vitamin D level at 3 months of the entire group was low (median: 32.1 IU/mL, IQR 13.6 IU/mL), and 24 of the total 63 (38.1%) infants had low levels of vitamin D (Table 2). How did you define a low vitamin D level?

Comparative analysis of effects of TDF and AZT on infant growth, renal function and bone health

Newborn body length, weight, and head circumference were significantly smaller in the AZT than TDF group (2-way ANOVA, adjusted for age (p=0.002, 0.047, and 0.049, respectively) (Figure 2 (A)-(C)). Furthermore, ALP was significantly higher in the TDF compared with the AZT group (p=0.007), whereas serum-creatinine, phosphorus and %TRP were not different (2-way ANOVA, p=0.505, 0.583, and 0.972, respectively) (Figure 2 (D)-(G)). The U-BMG level decreased with age in both treatment groups, and the prevalence of detectable U-BMG was not significantly different between the TDF and AZT groups [5/53 (9.4%) vs. 2/10 (20%) at 3 months (p=0.293), respectively (Figure 3A)]. There were no significant differences in the wrist radiographic scores for rickets between the two groups irrespective of age. Rapid improvement was observed with time after birth in the TDF group, and no abnormal findings were observed at 18 months of age. In comparison, one of the 5 infants ~~of~~ in the AZT group (20%) continued to have poor radiographic scores ~~with age~~ as they aged and the score was still abnormal at 18 months of age (Figure 3B).

Effects of confounding factors on infant growth, renal function and bone health

Multivariate linear regression analysis showed significant association between maternal TDF use and longer body length (estimate 2.52; 95% confidence interval (CI) 0.38-4.66, p=0.021). Apart from infant age, no other confounding ~~factor was~~ factors were associated with body length, weight, and head circumference (Table 3A). Multivariate linear regression analyses with the same independent variables as those used in the

models for physical parameters (Table 3B) showed a significant association between high serum alkaline phosphatase levels and maternal TDF [estimates 41.22; 95%CI: 6.98-75.45), p=0.019]. Overall, maternal TDF neither correlated with elevated serum creatinine, serum phosphorus, %TRP, radiographic score for rickets, nor with abnormal U-BMG. Interestingly, worse wrist radiographic score for rickets correlated significantly associated with maternal LPV/r, but not maternal TDF (estimate 0.55; 95%CI: 0.08-1.02, p=0.024).

what is this variable.

Post-hoc evaluation

Hemoglobin, hematocrit, erythrocyte count, and mean corpuscular volume (MCV) were measured during pregnancy in the HIV-infected mothers who had been managed at NHTD. Hemoglobin and hematocrit levels (measured in 31 mothers, TDF group: 23/53, AZT group: 8/10) were not significantly different between the AZT and TDF groups, while MCV was larger in the AZT than the TDF group (109.1 vs 95.7 fL, respectively, p=0.051, Table 1).

DISCUSSION

The main finding of the present study was that TDF use by pregnant Vietnamese women was not associated with infant growth impediment, renal dysfunction, or nutritional rickets, although it correlated with mild maternal renal tubular dysfunction. While we did not investigate the mechanisms of these changes in the present study, we postulate the following mechanisms. First, since growth is rapid in both late pregnancy and early infancy [7,18], preterm infants are at risk of metabolic bone

diseases in the presence of low supplies of phosphorus and calcium [19]. Similarly, TDF-associated maternal renal tubular dysfunction during late pregnancy can cause blunted fetal growth based on inadequate supply of phosphate [20]. In the present study, although TDF use during pregnancy was associated with significantly high maternal U-BMG levels, the maternal and infant %TRP levels were not significantly different between the TDF and AZT groups. These results suggest that TDF use by the Vietnamese pregnant mothers was not associated with significant renal loss of phosphorus ~~in order to cause infantile rickets.~~ Since high serum levels of ALP in the infants were associated with maternal TDF use, consistent with the findings in adult populations [4,21], maternal TDF exposure seems to influence bone health of infants at subclinical levels. Second, infants aged 3 months had below normal levels of 25-OH Vitamin D (<30 IU/mL) (in 38% of TDF group and 50% of AZT group). These data are consistent with a number of previous studies that demonstrated higher rates of vitamin D deficiency among Asians, compared with Caucasians, probably due to insufficient dietary intake [22,23]. Given ~~that nowadays~~ vitamin D deficiency rickets is the most common etiology of rickets [7] especially in Asian countries, the lower serum levels of 25-OH vitamin D seem to mask the impact of TDF-associated infant growth disturbance via renal loss of phosphate.

Surprisingly, our study identified AZT, rather than TDF, as the only significant risk factor for shorter infant body length. Various AZT toxic effects have been reported, including mitochondrial dysfunction with myopathy and cardiomyopathy [24], and bone marrow suppression [25]. Although reviews of ACTG 076 supported the safety of *in utero* exposure to AZT with respect to long-term infant

development/growth [26], serious concern has been raised about infant mitochondrial dysfunction [27,28] and cardiac toxicity [29]. Since AZT passes easily through the placenta, it confers high plasma concentrations in cord blood/peripheral circulation of infants [30]. Asian population with small body size could be vulnerable to AZT toxicity if exposed to AZT *in utero*. Unfortunately, blood samples were not obtained from the infants in the present study for ethical reasons and thus no hematological parameters, lactate levels, blood gases, or creatine kinase levels were measured to evaluate AZT toxicity in infants. Post-hoc analysis did not show any differences in hematologic parameters between TDF and AZT maternal groups. Further studies are needed to determine the effects of AZT and TDF use during pregnancy on infant growth.

LPV/r was used in 5 of the 10 (50%) AZT users but only in 2 of the 53 (3.8%) pregnant mothers of the TDF group. A number of well-designed randomized controlled studies reported significant association between maternal use of protease inhibitors, such as LPV/r, and fetal prematurity [31,32]. A more recent study concluded that the use of LPV/r was the most significant determinant of neonatal delayed growth [33]. Especially, protease inhibitors are reported to have adverse effects on bone health [6,34] through the activation of osteoclasts [35] or blockade of osteoblast differentiation [36]. Since bone ALP is the main isozyme in infants, the low levels of ALP observed in our study in the AZT group may partly explain the low osteoblast activity in LPV/r-exposed infants. Furthermore, given that perinatal and postnatal exposure to LPV/r, a potent cytochrome p450 inhibitor, causes clinically-

evident adrenal dysfunction [37], maternal exposure to LPV/r may have an adverse impact on infant growth or bone health.

Our study has certain limitations. First, the smaller sample size of AZT group limited the statistical power for appropriate comparison in renal/bone health markers, because TDF-containing regimens have been predominantly used for Vietnamese pregnant women in accordance with the 2013 launched WHO recommendation since then [1]. This limitation also prevented detailed analysis of the association of AZT and LPV/r with infant growth and bone health, although the main finding of the present study was the adverse effects of AZT and LPV/r. Second, infant growth should be compared using Z-scores to standardize the data for sex and race. To our knowledge, there are no established standard norms for physical growth of Vietnamese newborns and infants. In addition, normal values of tests of renal function, bone health, and serum levels of vitamin D are also not available. Although we tried to collect data of non-HIV mother-infant pairs to deal with the lack of normative data for Vietnamese, none of the enrolled non-HIV mother-infant pairs visited our center at the time of the study. In the present study, the higher percentage of male infants in the TDF group (34 of 53; 64%), compared with the AZT group (3 of 10; 30%), may explain the significant difference in body length between the two groups. However, in general, the difference in length between the sexes is only 1 cm from birth to 18 months of age [33], whereas the present study showed a larger than expected difference (49.2 vs. 46.0 cm at birth and 75.0 vs. 71.0cm at 18 months of age in the TDF and AZT group, respectively). Moreover, multivariate linear regression analysis showed that female sex was not associated with shorter length (p=0.825).

In conclusion, we have demonstrated in the present study that the use of TDF during pregnancy was not associated with harmful effects on neonatal and infant growth, renal function, or bone health, though it was associated with mild maternal renal tubular dysfunction in Vietnamese population. Maternal AZT and LPV/r showed significant association with infant growth and bone health, which requires further investigation.

Conflicts of Interest and Source of Funding:

E.K. has received honoraria from Gilead Sciences, ViiV Healthcare, and MSD. S.O. received honoraria from MSD, Janssen Pharmaceutical and Gilead Sciences, and research grants from MSD, ViiV Healthcare, Gilead Sciences. The remaining authors declare no conflict of interest.

This research was supported by the Japan Initiative for Global Research Network on Infectious Diseases from the Japan Agency for Medical Research and development, AMED. During the study period, antiretrovirals for Vietnamese patients were provided under the financial support of the U.S. president's emergency plan for AIDS relief (PEPFAR).

Acknowledgment

We thank the children, caretakers and staff of all facilities who participated in the present study, especially M. Sata and H. Nguyen (AIDS Clinical Center), D.T. Thuy and P.T. Tuyen (NHTD, Hanoi, Vietnam), and N.T. Trang (NHOG, Hanoi, Vietnam).

Author Contributions

Data curation: Hoai Dung Thi Nguyen, Ha Quan Do, Shoko Matsumoto, Moeko Nagai.

Formal analysis: Ei Kinai, Shoko Matsumoto.

Investigation: Ei Kinai, Hoai Dung Thi Nguyen, Ha Quan Do, Shoko Matsumoto, Moeko Nagai.

Project administration: Ei Kinai, Hoai Dung Thi Nguyen, Ha Quan Do.

Supervision: Junko Tanuma, Kinh Van Nguyen, Thach Ngoc Pham, Shinichi Oka.

Writing original draft: Ei Kinai

Figure Legends

Figure 1. Flow chart of the subject enrollment process. Sixty-seven HIV-infected and 10 HIV-negative pregnant women were recruited at the NHOG. Of the 57 TDF-treated mother-infant pairs, 4 pairs were excluded due to miscarriage (n=1), delivery at other hospitals (n=2), and twins (n=1). Although 3 of the 10 HIV-negative mothers were enrolled and provided maternal data and infant physical data at birth, none of the infants was brought to the center for further physical measurement and laboratory tests.

Figure 2. The dot plots provide comparisons of neonate/infant length (A), weight (B), and head circumference (C) between the TDF and AZT groups. The box-whisker plots provide comparisons of neonate/infant serum creatinine (D), serum phosphorus (E), serum alkaline phosphatase (F), and the percentage of tubular reabsorption of phosphate (G) between the two groups. Data were compared by 2-way ANOVA to adjust for the influence of age.

Figure 3.

Comparison of the prevalence of elevated urine β2-microglobulin (A) and abnormal radiographic wrist score for rickets (B) between the TDF and AZT groups. Data were compared by chi-square test.

References

1. WHO Guidelines Approved by the Guidelines Review Committee. Consolidated Guidelines on the Use of Antiretroviral Drugs for Treating and Preventing HIV Infection: Recommendations for a Public Health Approach. Geneva: World Health Organization, Copyright (c) World Health Organization 2013.; 2013.

2. Cooper RD, Wiebe N, Smith N, Keiser P, Naicker S, Tonelli M. Systematic review and meta-analysis: renal safety of tenofovir disoproxil fumarate in HIV-infected patients. Clin Infect Dis. 2010;51(5):496-505. Epub 2010/08/03. doi: 10.1086/655681. PubMed PMID: 20673002.

3. Sax PE, Wohl D, Yin MT, Post F, DeJesus E, Saag M, et al. Tenofovir alafenamide versus tenofovir disoproxil fumarate, coformulated with elvitegravir, cobicistat, and emtricitabine, for initial treatment of HIV-1 infection: two randomised, double-blind, phase 3, non-inferiority trials. Lancet (London, England). 2015;385(9987):2606-15. Epub 2015/04/22. doi: 10.1016/s0140-6736(15)60616-x. PubMed PMID: 25890673.

4. Kinai E, Hanabusa H. Progressive renal tubular dysfunction associated with long-term use of tenofovir DF. AIDS Res Hum Retroviruses. 2009;25(4):387-94. Epub 2009/04/14. doi: 10.1089/aid.2008.0202. PubMed PMID: 19361280.

5. Gallant JE, Moore RD. Renal function with use of a tenofovir-containing initial antiretroviral regimen. AIDS. 2009;23(15):1971-5. Epub 2009/08/22. doi: 10.1097/QAD.0b013e32832c96e9. PubMed PMID: 19696652; PubMed Central PMCID: PMCPMC3790472.

6. McComsey GA, Kitch D, Daar ES, Tierney C, Jahed NC, Tebas P, et al. Bone mineral density and fractures in antiretroviral-naive persons randomized to receive abacavir-lamivudine or tenofovir disoproxil fumarate-emtricitabine along with efavirenz or atazanavir-ritonavir: Aids Clinical Trials Group A5224s, a substudy of ACTG A5202. J Infect Dis. 2011;203(12):1791-801. Epub 2011/05/25. doi: 10.1093/infdis/jir188. PubMed PMID: 21606537; PubMed Central PMCID: PMCPMC3100514.

7. Wharton B, Bishop N. Rickets. Lancet. 2003;362(9393):1389-400. doi: 10.1016/s0140-6736(03)14636-3.

8. Van Rompay KK, Brignolo LL, Meyer DJ, Jerome C, Tarara R, Spinner A, et al. Biological effects of short-term or prolonged administration of 9-[2-(phosphonomethoxy)propyl]adenine (tenofovir) to newborn and infant rhesus macaques. Antimicrob Agents Chemother. 2004;48(5):1469-87. Epub 2004/04/24. doi: 10.1128/aac.48.5.1469-1487.2004. PubMed PMID: 15105094; PubMed Central PMCID: PMCPMC400569.

9. Siberry GK, Williams PL, Mendez H, Seage GR, 3rd, Jacobson DL, Hazra R, et al. Safety of tenofovir use during pregnancy: early growth outcomes in HIV-exposed uninfected infants. AIDS. 2012;26(9):1151-9. Epub 2012/03/03. doi: 10.1097/QAD.0b013e328352d135. PubMed PMID: 22382151; PubMed Central PMCID: PMCPMC3476702.

10. Nucleoside reverse transcriptase inhibitor backbones and pregnancy outcomes. AIDS. 2019;33(2):295-304. Epub 2018/12/19. doi: 10.1097/qad.0000000000002039. PubMed PMID: 30562172.

11. Pintye J, Langat A, Singa B, Kinuthia J, Odeny B, Katana A, et al. Maternal Tenofovir Disoproxil Fumarate Use in Pregnancy and Growth Outcomes among HIV-Exposed Uninfected Infants in Kenya. Infect Dis Obstet Gynecol. 2015;2015:276851. Epub 2016/01/30. doi: 10.1155/2015/276851. PubMed PMID: 26823647; PubMed Central PMCID: PMCPMC4707364.

12. Gibb DM, Kizito H, Russell EC, Chidziva E, Zalwango E, Nalumenya R, et al. Pregnancy and infant outcomes among HIV-infected women taking long-term ART with and without tenofovir in the DART trial. PLoS Med. 2012;9(5):e1001217. Epub 2012/05/23. doi: 10.1371/journal.pmed.1001217. PubMed PMID: 22615543; PubMed Central PMCID: PMCPMC3352861.

13. Siberry GK, Jacobson DL, Kalkwarf HJ, Wu JW, DiMeglio LA, Yogev R, et al. Lower Newborn Bone Mineral Content Associated With Maternal Use of Tenofovir Disoproxil Fumarate During Pregnancy. Clin Infect Dis. 2015;61(6):996-1003. Epub 2015/06/11. doi: 10.1093/cid/civ437. PubMed PMID: 26060285; PubMed Central PMCID: PMCPMC4551007.

14. Salvadori N, Fan B, Teeyasoontranon W, Ngo-Giang-Huong N, Phanomcheong S, Luvira A, et al. Maternal and Infant Bone Mineral Density 1 Year After Delivery in a Randomized, Controlled Trial of Maternal Tenofovir Disoproxil Fumarate to Prevent Mother-to-child Transmission of Hepatitis B Virus. Clin Infect Dis. 2019;69(1):144-6. Epub 2019/03/30. doi: 10.1093/cid/ciy982. PubMed PMID: 30924492; PubMed Central PMCID: PMCPMC6579954.

15. Kourtis AP, Wiener J, Wang L, Fan B, Shepherd JA, Chen L, et al. Tenofovir Disoproxil Fumarate Use during Pregnancy and Infant Bone Health: the Tenofovir in Pregnancy Pilot Study. Pediatr Infect Dis J. 2018;37(11):e264-e8. Epub 2018/08/02. doi: 10.1097/inf.0000000000002152. PubMed PMID: 30067600.

16. Nishijima T, Komatsu H, Gatanaga H, Aoki T, Watanabe K, Kinai E, et al. Impact of small body weight on tenofovir-associated renal dysfunction in HIV-infected patients: a retrospective cohort study of Japanese patients. PloS ONE. 2011;6(7):e22661. Epub 2011/07/30. doi: 10.1371/journal.pone.0022661. PubMed PMID: 21799928; PubMed Central PMCID: PMCPMC3143186.

17. Thacher TD, Fischer PR, Pettifor JM, Lawson JO, Manaster BJ, Reading JC. Radiographic scoring method for the assessment of the severity of nutritional rickets. J Trop Pediatr. 2000;46(3):132-9. Epub 2000/07/14. doi: 10.1093/tropej/46.3.132. PubMed PMID: 10893912.

18. Bishop N. Bone disease in preterm infants. Arch Dis Child. 1989;64(10 Spec No):1403-9. Epub 1989/10/01. doi: 10.1136/adc.64.10_spec_no.1403. PubMed PMID: 2686555; PubMed Central PMCID: PMCPMC1590102.

19. Carpenter TO, Shaw NJ, Portale AA, Ward LM, Abrams SA, Pettifor JM. Rickets. Nat Rev Dis Prim. 2017;3:17101. Epub 2017/12/22. doi: 10.1038/nrdp.2017.101. PubMed PMID: 29265106.

20. Kinai E, Hosokawa S, Gomibuchi H, Gatanaga H, Kikuchi Y, Oka S. Blunted fetal growth by tenofovir in late pregnancy. AIDS. 2012;26(16):2119-20. Epub 2012/10/12. doi: 10.1097/QAD.0b013e328358ccaa. PubMed PMID: 23052356.

21. Fux C, Rauch A, Simcock M, Bucher H, Hirschel B, Opravil M, et al. Tenofovir Use Is Associated with an Increase in Serum Alkaline Phosphatase in the Swiss HIV Cohort Study. Antivir Ther. 2008;13:1077-82.

22. Lawson M, Thomas M. Vitamin D concentrations in Asian children aged 2 years living in England: population survey. BMJ. 1999;318(7175):28. Epub 1999/01/05. doi: 10.1136/bmj.318.7175.28. PubMed PMID: 9872879; PubMed Central PMCID: PMCPMC27672.
23. Chen YH, Fu L, Hao JH, Wang H, Zhang C, Tao FB, et al. Influent factors of gestational vitamin D deficiency and its relation to an increased risk of preterm delivery in Chinese population. Sci Rep. 2018;8(1):3608. Epub 2018/02/28. doi: 10.1038/s41598-018-21944-3. PubMed PMID: 29483547; PubMed Central PMCID: PMCPMC5827025.
24. Dalakas MC, Illa I, Pezeshkpour GH, Laukaitis JP, Cohen B, Griffin JL. Mitochondrial myopathy caused by long-term zidovudine therapy. N Engl J Med. 1990;322(16):1098-105. Epub 1990/04/19. doi: 10.1056/nejm199004193221602. PubMed PMID: 2320079.
25. Aurpibul L, Puthanakit T, Sirisanthana T, Sirisanthana V. Haematological changes after switching from stavudine to zidovudine in HIV-infected children receiving highly active antiretroviral therapy. HIV Med. 2008;9(5):317-21. Epub 2008/03/12. doi: 10.1111/j.1468-1293.2008.00560.x. PubMed PMID: 18331562.
26. Sperling RS, Shapiro DE, McSherry GD, Britto P, Cunningham BE, Culnane M, et al. Safety of the maternal-infant zidovudine regimen utilized in the Pediatric AIDS Clinical Trial Group 076 Study. AIDS. 1998;12(14):1805-13. Epub 1998/10/29. doi: 10.1097/00002030-199814000-00012. PubMed PMID: 9792381.
27. Blanche S, Tardieu M, Rustin P, Slama A, Barret B, Firtion G, et al. Persistent mitochondrial dysfunction and perinatal exposure to antiretroviral nucleoside analogues. Lancet. 1999;354(9184):1084-9. Epub 1999/10/06. doi: 10.1016/s0140-6736(99)07219-0. PubMed PMID: 10509500.
28. Barret B, Tardieu M, Rustin P, Lacroix C, Chabrol B, Desguerre I, et al. Persistent mitochondrial dysfunction in HIV-1-exposed but uninfected infants: clinical screening in a large prospective cohort. AIDS. 2003;17(12):1769-85. Epub 2003/08/02. doi: 10.1097/00002030-200308150-00006. PubMed PMID: 12891063.
29. Lipshultz SE, Easley KA, Orav EJ, Kaplan S, Starc TJ, Bricker JT, et al. Absence of cardiac toxicity of zidovudine in infants. Pediatric Pulmonary and Cardiac Complications of Vertically Transmitted HIV Infection Study Group. N Engl J Med. 2000;343(11):759-66. Epub 2000/09/14. doi: 10.1056/nejm200009143431102. PubMed PMID: 10984563.
30. Kinai E, Kato S, Hosokawa S, Sadatsuki M, Gatanaga H, Kikuchi Y, et al. High Plasma Concentrations of Zidovudine (AZT) Do Not Parallel Intracellular Concentrations of AZT-Triphosphates in Infants During Prevention of Mother-to-Child HIV-1 Transmission. J Acquir Immune Defic Syndr. 2016;72(3):246-53. Epub 2016/02/10. doi: 10.1097/qai.0000000000000950. PubMed PMID: 26859826.
31. Powis KM, Kitch D, Ogwu A, Hughes MD, Lockman S, Leidner J, et al. Increased risk of preterm delivery among HIV-infected women randomized to protease versus nucleoside reverse transcriptase inhibitor-based HAART during pregnancy. J Infect Dis. 2011;204(4):506-14. Epub 2011/07/28. doi: 10.1093/infdis/jir307. PubMed PMID: 21791651; PubMed Central PMCID: PMCPMC3144169.
32. Patel K, Van Dyke RB, Mittleman MA, Colan SD, Oleske JM, Seage GR, 3rd,

et al. The impact of HAART on cardiomyopathy among children and adolescents perinatally infected with HIV-1. AIDS. 2012;26(16):2027-37. Epub 2012/07/12. doi: 10.1097/QAD.0b013e3283578bfa. PubMed PMID: 22781228; PubMed Central PMCID: PMCPMC3513344.

33. Blanche S, Tylleskar T, Peries M, Kankasa C, Engebretsen I, Meda N, et al. Growth in HIV-1-exposed but uninfected infants treated with lopinavir-ritonavir versus lamivudine: a secondary analysis of the ANRS 12174 trial. Lancet HIV. 2019;6(5):e307-e14. Epub 2019/03/01. doi: 10.1016/s2352-3018(18)30361-8. PubMed PMID: 30814028.

34. Kinai E, Nishijima T, Mizushima D, Watanabe K, Aoki T, Honda H, et al. Long-term use of protease inhibitors is associated with bone mineral density loss. AIDS Res Hum Retroviruses. 2014;30(6):553-9. Epub 2014/02/06. doi: 10.1089/aid.2013.0252. PubMed PMID: 24494779.

35. Modarresi R, Xiang Z, Yin M, Laurence J. WNT/beta-catenin signaling is involved in regulation of osteoclast differentiation by human immunodeficiency virus protease inhibitor ritonavir: relationship to human immunodeficiency virus-linked bone mineral loss. Am J Pathol. 2009;174(1):123-35. Epub 2008/12/20. doi: 10.2353/ajpath.2009.080484. PubMed PMID: 19095956; PubMed Central PMCID: PMCPMC2631325.

36. Hirakawa H, Gatanaga H, Ochi H, Fukuda T, Sunamura S, Oka S, et al. Antiretroviral Therapy Containing HIV Protease Inhibitors Enhances Fracture Risk by Impairing Osteoblast Differentiation and Bone Quality. J Infect Dis. 2017;215(12):1893-7. Epub 2017/05/20. doi: 10.1093/infdis/jix246. PubMed PMID: 28525596.

37. Simon A, Warszawski J, Kariyawasam D, Le Chenadec J, Benhammou V, Czernichow P, et al. Association of prenatal and postnatal exposure to lopinavir-ritonavir and adrenal dysfunction among uninfected infants of HIV-infected mothers. JAMA. 2011;306(1):70-8. Epub 2011/07/07. doi: 10.1001/jama.2011.915. PubMed PMID: 21730243.

33. World Health Organization: The WHO child growth standards**.** Geneva, World Health Organization. 2009

Table 1. Clinical characteristics and laboratory test results of pregnant women at 32-36 weeks of gestation.

	Total (n=63)	TDF (n=53)	AZT (n=10)	P value
Age (years), median (IQR)	32 (8.0)	32 (6.1)	32 (8.7)	0.805
Body weight (kg), median (IQR)	57.3 (11.0)	57.5 (12.3)	57.3 (6.5)	0.601
Gestational complications, n (%)	2 (3.2)	1 (1.9)	1 (10)	0.382
Current Smoking, n (%)	1 (1.6)	1 (1.9)	0 (0)	n.a.
Alcohol use, n (%)	0 (0)	0 (0)	0 (0)	n.a.
CD4 cell count (median, IQR)	298 (201)	394 (191)	442 (205)	0.689
HIV-RNA undetectable (<20) (n, %)	49 (78)	41 (77)	8 (80)	0.854
Alkaline phosphatase (IU/L), median (IQR)	102 (33)	104 (33)	84 (40)	0.162
Creatinine (mg/dL), median (IQR)	0.59 (0.16)	0.59 (0.15)	0.53 (0.19)	0.276
Calcium (mg/dL), median (IQR)	8.8 (0.5)	8.7 (0.4)	8.8 (0.4)	0.316
Phosphorus (mg/dL), median (IQR)	3.3 (0.6)	3.1 (0.9)	3.6 (0.5)	0.358
U=BMG (μg/L), median (IQR)	2755 (5222)	3060 (5915)	581 (3322)	<0.001
Detectable U-BMG (>252 μg/L), n (%)	52 (83)	47 (89)	5 (50)	<0.001
%TRP (%), median (IQR)	92.2 (6.2)	91.5 (6.1)	93.8 (6.1)	0.241
Low %TRP (<90%), n (%)	22 (34.9)	20 (38.5)	2 (20.0)	0.309
Very low %TRP (<80%), n (%)	0 (0)	0 (0)	0 (0)	n.a.
*Hemoglobin (g/dL), median (IQR)	12.4 (1.4)	12.6 (1.7)	12.1 (0.8)	0.260
*Hematocrit (%), median (IQR)	36.9 (4.5)	37.3 (4.6)	35.3 (1.1)	0.214
*Mean corpuscular volume (fL), median (IQR)	97.4 (11.2)	95.7 (7.2)	109.3 (20.5)	0.051
History of AIDS-defining illness, n (%)	0 (0)	0 (0)	0 (0)	n.a.
ART initiation				
Prior to pregnancy, n (%)	56 (89)	47 (89)	9 (90)	0.903
During pregnancy, n (%)	7 (11)	6 (11)	1 (10)	
ART duration (months), median (IQR)	29 (51.7))	28 (55.3)	47 (53.8)	0.155
History of TDF use, n (%)	57 (90)	53 (100)	4 (40)	n.a.
TDF duration (months), median (IQR)	22 (38.3)	26 (48.2)	3 (28.3)	0.235
Key drug during pregnancy				
EFV, n (%)	49 (77.8)	49 (92.4)	0 (0)	n.a.
NVP, n (%)	7 (11.1)	2 (3.8)	5 (50)	<0.001
LPV/r, n (%)	7 (11.1)	2 (3.8)	5 (50)	<0.001

IQR: interquartile range, TDF; tenofovir disoproxil fumarate, AZT; zidovudine, U-BMG; urine-β2-microglobulin, %TRP; the percentage of renal tubular reabsorption of phosphate, EFV; efavirenz, NVP; nevirapine, LPV/r; lopinavir-ritonavir (Kaletra)

P value was determined by chi-square test of t-test in comparison of TDF and AZT group.

*Blood samples for hemoglobin, hematocrit, and mean corpuscular volume were collected

from 31/63 participants (23 of TDF-treated mothers and 8 of AZT-treated mothers) for post-hoc analysis.

Table 2. Baseline clinical characteristics of infants

	Total (n=63)	TDF (n=53)	AZT (n=10)	P value	Non-HIV (n=2)
Sex (male): n, (%)	37 (58.7)	34 (64.2)	3 (30)	0.129	1 (33)
Gestational age (week), median (IQR)	39 (1.1)	39 (1)	39 (1.3)	0.462	39 (n.a.)
Birth length (cm), median (IQR)	48.0 (2.3)	49.2 (1.7)	46.0 (8.5)	0.061	52.5 (n.a.)
Birth weight (g), median (IQR)	3100 (500)	3200 (550)	2900 (750)	0.165	3400 (n.a.)
Birth head circumference (cm), median (IQR)	34.0 (2.0)	34 (1.8)	32.5 (5.6)	<0.001	35.5 (n.a.)
APGAR score (1 min), median (IQR)	9 (1)	9 (1)	9 (1)	NS	7 (n.a.)
APGAR score (5 min), median (IQR)	10 (0)	10 (0)	10 (0)	NS	8.5 (n.a.)
Perinatal complication, n (%)	0 (0)	0 (0)	0 (0)	NS	1 (50)
Congenital abnormalities, n (%)	0 (0)	0 (0)	0 (0)	NS	0 (0)
Nutrition (breastfeeding), n (%)	0 (0)	0 (0)	0 (0)	NS	1 (50)
Postpartum antiretroviral					
Zidovudine, n (%)	0 (0)	0 (0)	0 (0)	NS	
Nevirapine, n (%)	63 (100)	53 (100)	10 (100)	NS	
Duration of postpartum antiretrovirals (wks), median (IQR)	6 (0)	6 (0)	6 (0)	NS	
25-OH Vitamin D at 3 months (IU/mL), median (IQR)	32.1 (13.6)	32.3 (13.8)	27.6 (15.6)	0.411	n.a.
Low 25-OH Vitamin D at 3 months (<30 IU/mL), n (%)	24 (38.1)	19 (35.8)	5 (50)	0.428	n.a.
Deficient (<20 IU/mL), n (%)	6 (9.5)	6 (11.3)	0 (0)	0.316	n.a.
Insufficient (20-30 IU/mL), n (%)	18 (28.6)	13 (24.5)	5 (50)	0.242	n.a.

IQR: interquartile range, TDF; tenofovir disoproxil fumarate, AZT; zidovudine, n.a.: not available, NS: not significant

Table 3A. Results of multivariate regression analysis for infant body length, weight, and head circumference.

	Length		Weight		Head Circumference	
	Estimate (95% CI)	P value	Estimate (95% CI)	P value	Estimate (95% CI)	P value
Maternal TDF (yes or no)	**2.52 (0.38 to 4.66)**	**0.021**	130.1 (-393.1 to 653.3)	0.624	0.47 (-0.81 to 1.74)	0.470
Maternal LPV/r (yes or no)	0.61 (-1.84 to 3.06)	0.626	-75.6 (-675.4 to 524.2)	0.804	-0.34 (-1.79 to 1.12)	0.643
Gestational week at birth (per 1 week increase)	0.20 (-1.69 to 1.35)	0.542	33.2 (-127.0 to 193.4)	0.683	-0.16 (-0.55 to 0.24)	0.434
Infant sex (male =1, female =0)	-0.17 (-0.004 to 0.007)	0.825	-28.4 (-398.0 to 341.1)	0.879	-0.54 (-1.45 to 0.36)	0.238
Infant age (per 1 month increase)	1.83 (1.73 to 1.93)	<0.001	-149.0 (-174.7 to -123.3)	<0.001	0.75 (0.68 to 0.81)	<0.001

Table 3B. Results of multivariate regression analysis for infant body length, weight, and head circumference.

	Maternal TDF (yes/no)	Maternal LPV/r (yes/no)	Gestational age (/week increase)	Infant sex (m=1, f=0)	Infant age (/month increase)
Serum creatinine					
Estimate (95% CI)	0.03 (-0.02 to 0.07)	0.03 (-0.03 to 0.08)	0.009 (-0.005 to 0.023)	0.01 (-0.02 to 0.04)	**0.005 (0.003 to 0.008)**
P value	0.211	0.315	0.225	0.541	**<0.001**
Serum phosphorus					
Estimate (95% CI)	-0.05 (-0.39 to 0.29)	-0.13 (-0.53 to 0.26)	-0.04 (-0.15 to 0.07)	0.10 (-0.10 to 0.36)	**-0.08 (-0.10 to -0.06)**
P value	0.773	0.507	0.474	0.427	**<0.001**
Serum alkaline phosphatase					
Estimate (95% CI)	**41.22 (6.98 to 75.45)**	-14.42 (-54.01 to 25.17)	7.92 (-3.04 to 18.87)	5.64 (-20.02 to 31.3)	**-5.34 (-7.25 to -3.42)**
P value	**0.019**	0.472	0.155	0.664	**<0.001**
%TRP					
Estimate (95% CI)	0.09 (-4.93 to 5.12)	1.05 (-4.77 to 6.86)	**-1.68 (-3.29 to -0.07)**	-0.71 (-4.48 to 3.06)	**-0.33 (-0.61 to -0.53)**
P value	0.971	0.722	**0.041**	0.709	**0.020**
Wrist radiographic scores					
Estimate (95% CI)	0.15 (-0.26 to 0.57)	**0.55 (0.08 to 1.02)**	0.08 (-0.05 to 0.21)	-0.06 (-0.37 to 0.25)	**-0.05 (-0.08 to -0.03)**
P value	0.463	**0.024**	0.242	0.709	**<0.001**
Detectable U-BMG					
Odds ratio (95% CI)	0.56 (0.11 to 2.80)	0.37 (0.04 to 3.92)	0.96 (0.90 to 1.03)	1.21 (0.35 to 4.13)	0.96 (0.87 to 1.06)
P value	0.480	0.407	0.281	0.763	0.391

TDF: tenofovir disoproxil fumarate, LPV/r: the combination of lopinavir and ritonavir (Kaletra)

Differences in U-BMG were tested by logistic multivariate analysis, with U-BMG detectable level (>252 µg/L) set as the independent variable.

List and legends of Supplementary Figures.

Supplementary Figure 1.

Comparison of the prevalence of elevated urine β2-microglobulin (A) and abnormal radiographic wrist score for rickets (B) between the TDF and AZT groups. Data were compared by chi-square test.

Figures 1-3 Click here to access/download;Figure;TDF and rickets-Figures 210215.pptx

Figure 1

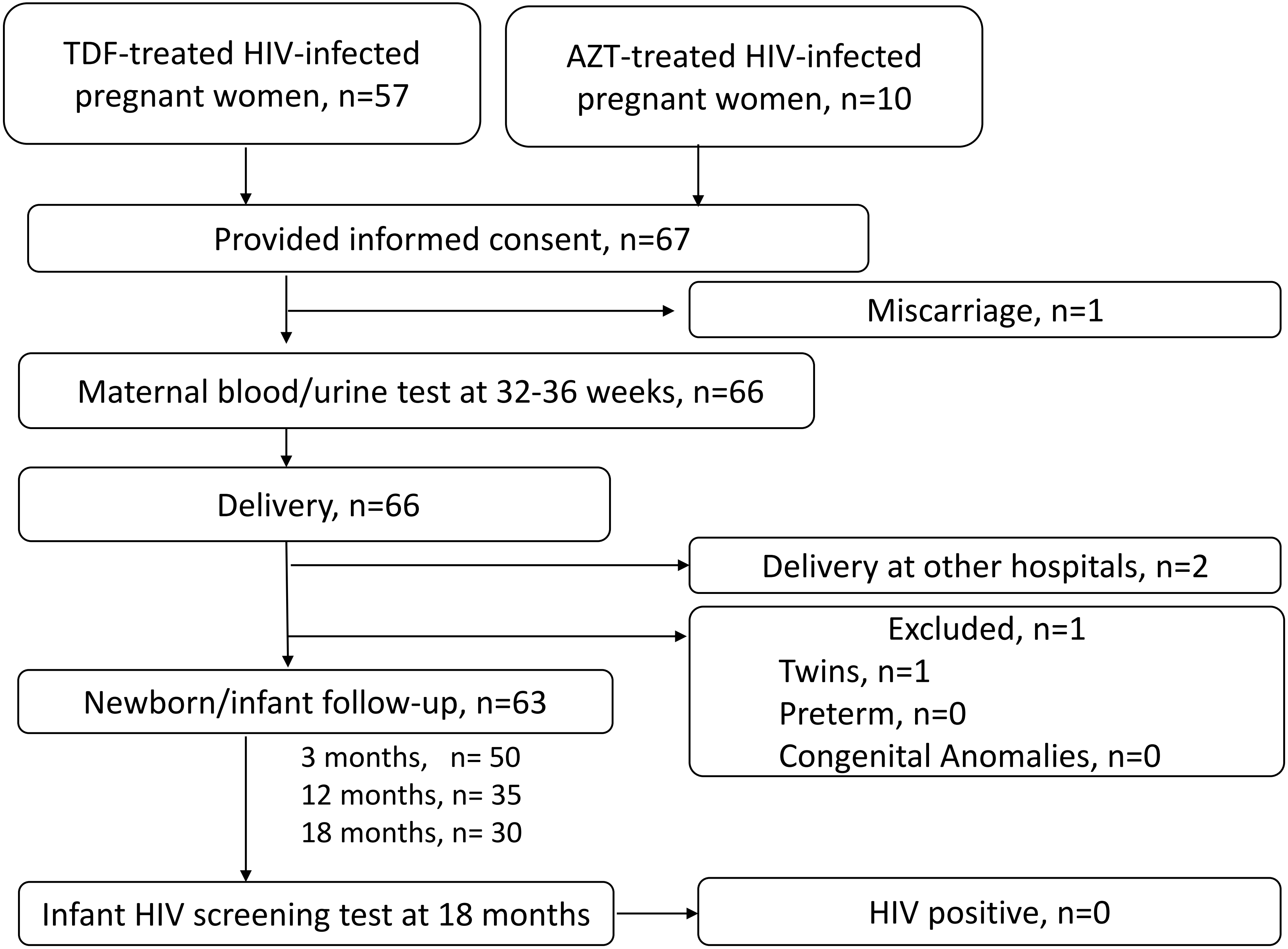

Figure 2

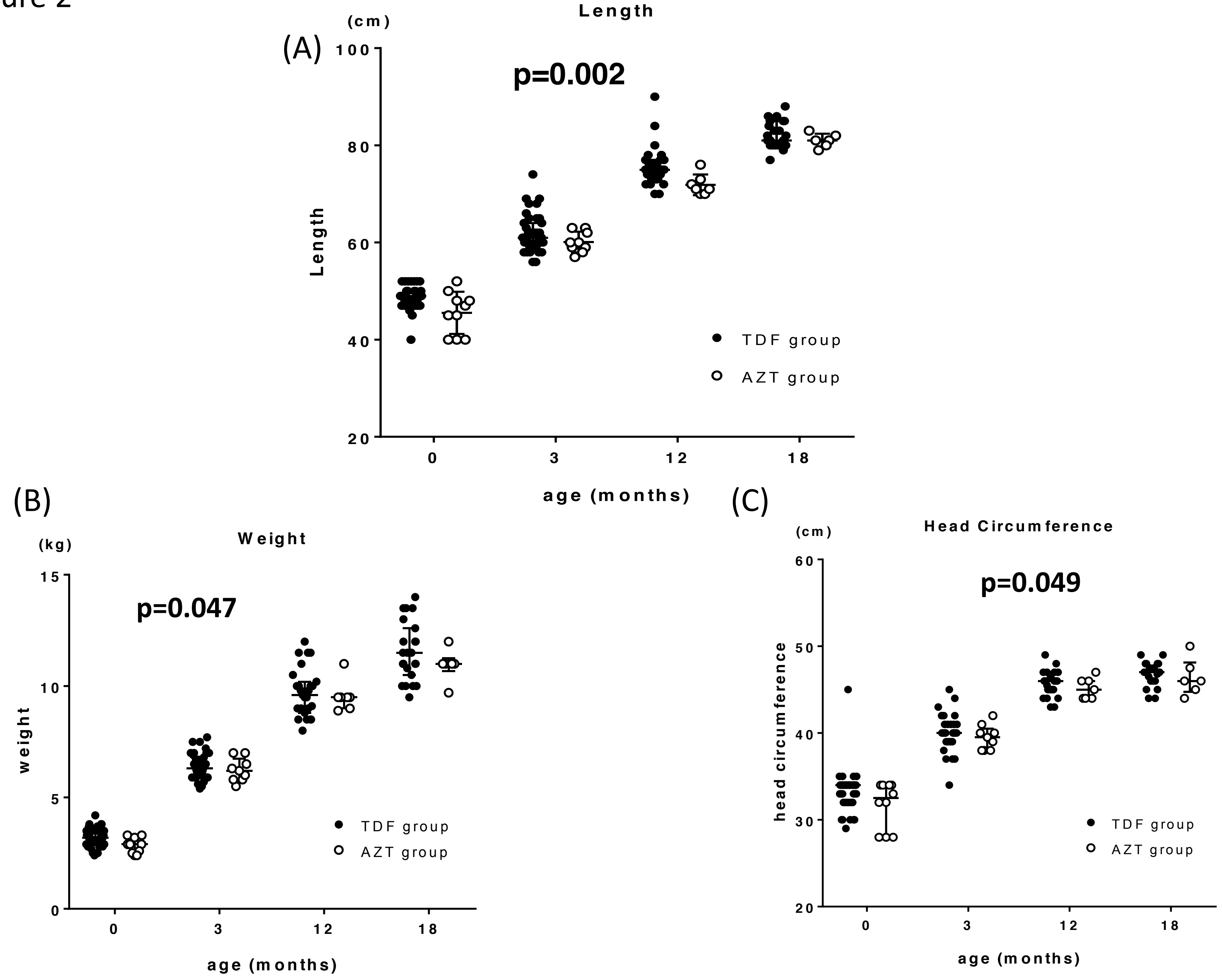
(A)
Length
(cm)
p=0.002
Length
100
80
60
40
20
0
3
12
18
age (months)
TDF group
AZT group
(B)
Weight
(kg)
p=0.047
weight
15
10
5
0
0
3
12
18
age (months)
TDF group
AZT group
(C)
Head Circumference
(cm)
p=0.049
head circumference
60
50
40
30
20
0
3
12
18
age (months)
TDF group
AZT group

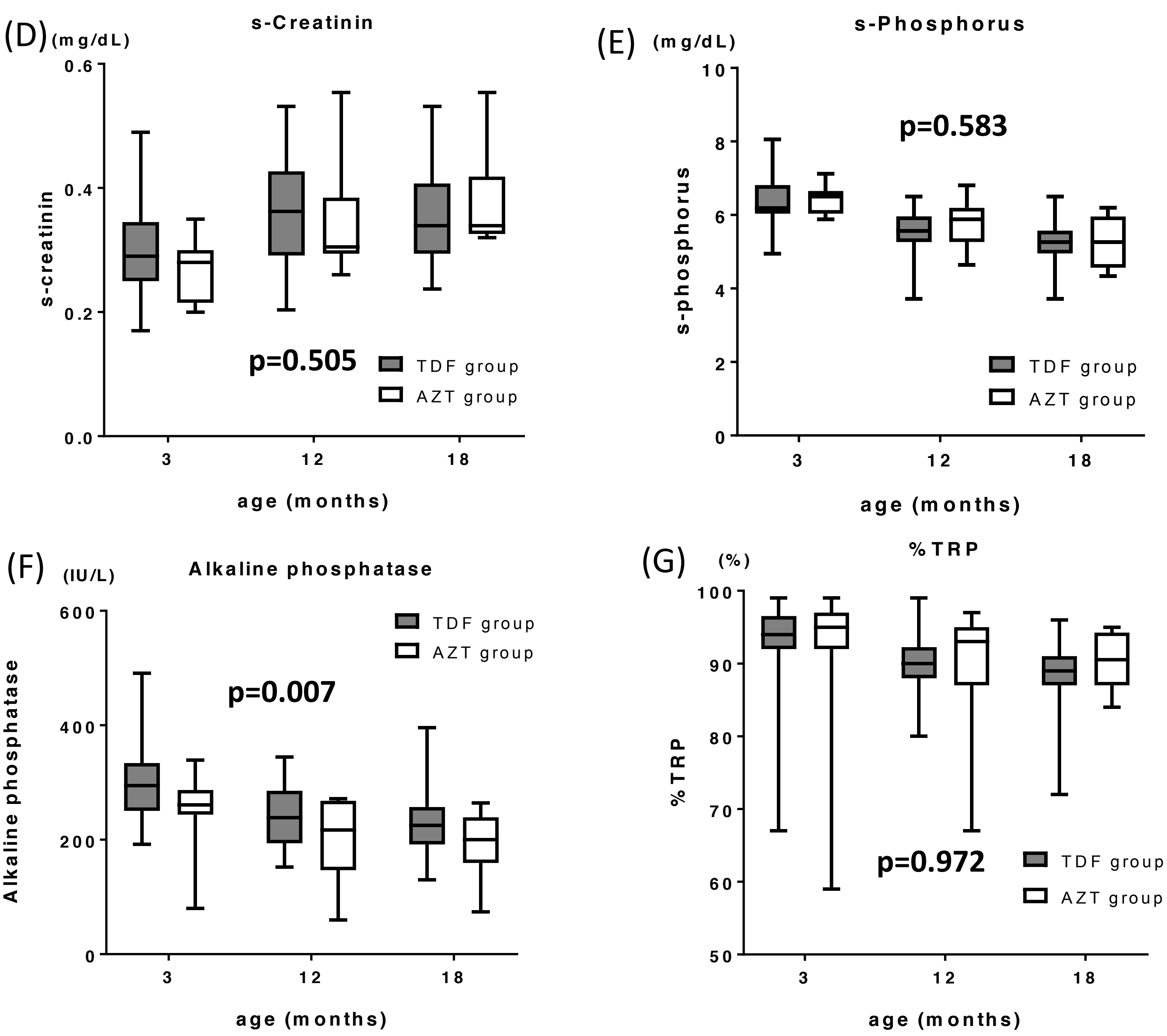

(D)
s-Creatinin
(mg/dL)
s-creatinin
0.0
0.2
0.4
0.6
3
12
18
age (months)
p=0.505
TDF group
AZT group
(E)
s-Phosphorus
(mg/dL)
s-phosphorus
0
2
4
6
8
10
3
12
18
age (months)
p=0.583
TDF group
AZT group
(F)
Alkaline phosphatase
(IU/L)
Alkaline phosphatase
0
200
400
600
3
12
18
age (months)
p=0.007
TDF group
AZT group
(G)
%TRP
(%)
%TRP
50
60
70
80
90
100
3
12
18
age (months)
p=0.972
TDF group
AZT group

Figure 3

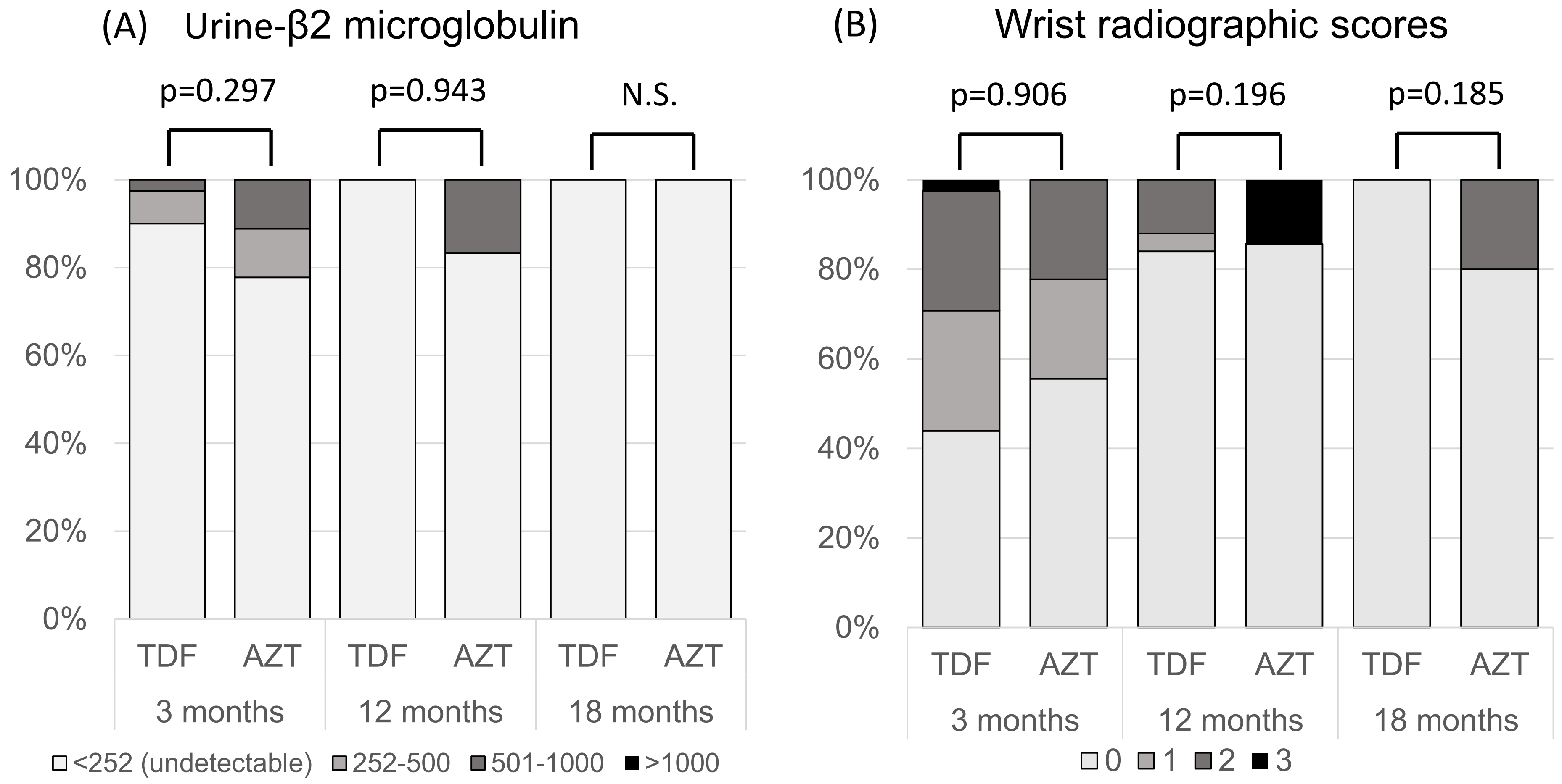

(A) Urine-β2 microglobulin
p=0.297
p=0.943
N.S.
100%
80%
60%
40%
20%
0%
TDF
AZT
TDF
AZT
TDF
AZT
3 months
12 months
18 months
<252 (undetectable)
252-500
501-1000
>1000
(B) Wrist radiographic scores
p=0.906
p=0.196
p=0.185
100%
80%
60%
40%
20%
0%
TDF
AZT
TDF
AZT
TDF
AZT
3 months
12 months
18 months
0
1
2
3
